# Kayexalate-Induced Esophageal Ulceration in a Patient with Decompensated Cirrhosis: A Review of the Literature

**DOI:** 10.1155/2021/8831814

**Published:** 2021-02-26

**Authors:** Kishore Kumar, Harish Patel, Muhammad Saad, Ahmed Baiomi, Anil Dev

**Affiliations:** ^1^Division of Gastroenterology, BronxCare Hospital Center a Clinical Affiliate of Mt Sinai Health Systems and Academic Affiliate of Icahn School of Medicine, Bronx, NY 10457, USA; ^2^Department of Medicine, BronxCare Hospital Center a Clinical Affiliate of Mt Sinai Health Systems and Academic Affiliate of Icahn School of Medicine, Bronx, NY 10457, USA

## Abstract

Hyperkalemia is one of the most common electrolyte abnormalities encountered in clinical practice. The treatment of hyperkalemia includes removal of excess potassium from the body using cation exchange resins, e.g., sodium polystyrene sulfonate (Kayexalate) is one of the most practiced modalities in clinical medicine. Colonic mucosal necrosis and perforation are the serious gastrointestinal side effects associated with sodium polystyrene sulfonate (SPS) use, which have been reported with or without concomitant use of sorbitol. However, the catastrophic bleeding esophageal ulcer has been rarely described in our literature search. Due to the risk of colonic necrosis, the FDA has issued a warning to avoid concomitant sorbitol use with Kayexalate. We present an individual with acute hematemesis due to bleeding esophageal ulcer immediately after treatment with Kayexalate therapy. Though the exact mechanism by which Kayexalate causes esophageal ulcer to be elucidated, nonetheless it is worthwhile to be vigilant about its potential adverse effects. Our case highlights the rare but certainly the life-threatening complication of Kayexalate therapy.

## 1. Case Presentation

**Figure 1:**
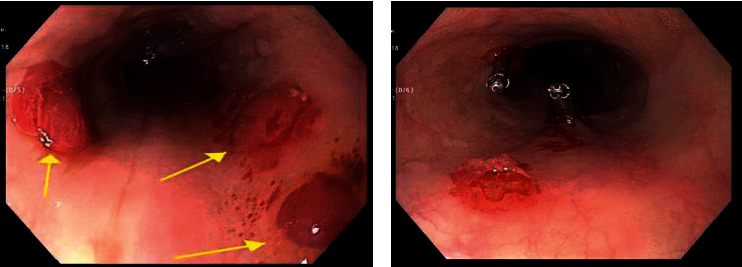
(a) Endoscopic view of esophageal ulcer with active oozing. (b) Endoscopic treatment of esophageal ulcer with hemostatic clips.

A 63-year-old Hispanic man was following nephrology clinic for the management of the nephrotic syndrome. He was recalled to the emergency department (ED) by the renal team due to an elevated potassium level of 6.1 mg/dl on his routine basic metabolic panel. On arrival to the ED, he was afebrile with normal heart rate and blood pressure. His physical examination included a distended nontender abdomen and 2+ pitting edema of lower extremities. The rest of the physical examination included cardiovascular, pulmonary, and neurologic examination which were within normal limits. The etiology of the nephrotic syndrome was not certain; however, the workup was unremarkable for some of the common etiologies which could cause nephrotic syndrome (Table 1). He was also known to the gastrointestinal (GI) team for his known history of decompensated liver cirrhosis due to alcohol use and small nonbleeding esophageal varices on endoscopy for which he was being treated with nonselective beta-blocker as primary prophylactic therapy. Previously, he had undergone multiple endoscopic procedures for evaluation of anemia and obscure small bowel bleeding. The last endoscopic procedure was performed 3 weeks before this admission, and he was noticed to have nonbleeding small esophageal varices, otherwise unremarkable small bowel enteroscopy. On arrival to the ED, his repeat blood chemistry showed an elevated potassium level of 6.6 mg/dl for which he was treated oral sodium polystyrene (Kayexalate suspension) of 30 mg in addition to intravenous (I/V) dextrose, calcium gluconate, and insulin. Other pertinent laboratory results are shown in Table 2. Thirty to forty-five minutes after oral sodium polystyrene (Kayexalate) therapy, the patient developed a large episode of hematemesis. He was immediately treated with I/V octreotide, I/V proton-pump inhibitor, I/V antibiotics, and a single dose of prokinetic agent. He underwent emergent hemodialysis for refractory hyperkalemia and acute kidney injury after inserting an emergent femoral vein Shiley catheter. Subsequently, he required endotracheal intubation for airway protection due to recurrent episodes of hematemesis. The emergent endoscopy was performed after the correction of the potassium level. The esophagogastroduodenoscopy (EGD) identified multiple round, sharply demarcated, discrete, esophageal ulcers with mucosal oozing (Figure 1(a)) and small nonbleeding esophageal varices again seen. The two of the esophageal ulcers were actively oozing, to achieve the hemostasis, and two hemostatic clips were deployed with the cessation of bleeding (Figure 1(b)). There was no evidence of bleeding lesions in the stomach and duodenum. The esophageal ulcer biopsies were not taken due to the concern of causing iatrogenic bleeding in the presence of coagulopathy. He had no evidence of recurrent gastrointestinal bleeding. His hospital course was further complicated with the development of septic shock due to pneumonia requiring multiple vasopressor support, disseminated intravascular coagulation (DIC), poor recovery of renal functions requiring renal replacement therapy, and multiorgan failure (MOF). Given poor recovery and prognosis, the family signed advance directives of do not resuscitate (DNR). Eventually, he sustained cardiac arrest and died. The patient family decided against autopsy; thus, histologic confirmation of esophageal abnormalities identified on endoscopic examination could not be attained.

## 2. Introduction

Hyperkalemia is one of the most common electrolyte abnormalities encountered in clinical practice. Depending on the severity of hyperkalemia, it has the potential to cause life-threatening arrhythmias leading to sudden cardiac arrest and death. Therefore, the clinicians remained very vigilant in treating hyperkalemia. The prevalence of hyperkalemia is high in an individual with comorbidities of chronic kidney disease, diabetes mellitus, hypertension leading to impaired renal functions, and use of a specific group of medications that are well known to cause hyperkalemia such as angiotensin-converting enzyme or angiotensin receptor blocker.

The treatment of hyperkalemia includes temporizing measures to shift excessive potassium into the cells with the use of insulin, calcium, bicarbonate, beta-2 agonist, and dextrose. In addition to the above, the medications which could cause hyperkalemia should be avoided. However, the definitive therapeutic modalities should be used to remove unwanted potassium from the body which include the use of loop diuretics, cation exchange resins, e.g., sodium polystyrene sulfonate (Kayexalate), and, in refractory cases, with the help of dialysis which remained the most effective to eliminate potassium. The serious gastrointestinal side effects of sodium polystyrene sulfonate (SPS) have been reported with or without concomitant use of sorbitol including colonic perforation.

Many other gastrointestinal adverse effects have been reported from Kayexalate use including anorexia, nausea, vomiting, constipation, fecal impaction, and intestinal obstruction [1]. However, based on our literature review, the bleeding esophageal ulcer requiring endoscopic intervention has not been reported previously. We mentioned a patient with acute hematemesis due to bleeding esophageal ulcer developed immediately after treatment with Kayexalate therapy in the emergency department. On upper endoscopy, the esophageal ulcers were well demarcated with active mucosal oozing being noted. To stop the bleeding and achieve hemostasis, two hemostatic clips were successfully placed.

## 3. Discussion

It is particularly important to ensure that the potassium level is not erroneous due to inappropriate sample collection, hemolysis, thrombocytosis, and leukocytosis [2]. Kayexalate was first introduced in 1950, and the name “Kayexalate” was given to the powdered form of sodium polystyrene sulfonate (SPS). Randomized controlled trial has shown that Kayexalate is more effective when given chronically; however, the benefit is at the expense of commonly experienced gastrointestinal side effects as mentioned above. Sodium bicarbonate therapy is not a preferred approach for acute management of hyperkalemia except in patients with metabolic acidosis or normal renal functions [2]. Kayexalate-induced gastrointestinal side effects are more common in patients with underlying kidney disease, s/p solid organ transplantation, or immediate postoperative period [3].

It appears that existing mucosal ischemia predisposes gastrointestinal mucosa to Kayexalate/sorbitol injury. The presence of uremia, elevated renin level, and hypotension will cause an increase in the release of angiotensin which subsequently gives rise to vasoconstriction. Although renal failure has been very well documented in the literature precipitating Kayexalate-induced gastrointestinal side effects, the effects of end-stage liver disease or portal hypertension need to be elucidated.

Kayexalate exchanges sodium for potassium, calcium, ammonium, and magnesium. When ingested orally, the sulfonate group of SPS is bound to the hydrogen ion at acidic pH, therefore unable to bind potassium. Later, when it travels through the small intestine and reaches the large intestine, the hydrogen ions are replaced with potassium. The most potassium exchange takes place in the colonic region due to a higher concentration of potassium in the colon. The tendency of SPS to cause intestinal obstruction is demonstrated by the fact that it binds to calcium in the gastrointestinal lumen and forms a concrete material; another possible mechanism is a predilection of resins to swells once contact with water in the gastrointestinal tract causes severe constipation and fecal impaction [4]. To decrease the risk of bowel obstruction and perforation, the SPS is then mixed with sorbitol which functions as a cathartic agent and causes osmotic diarrhea [5]. However, the U.S. Food and Drug Administration (FDA) has issued a warning to avoid Kayexalate administration with sorbitol due to the risk of colonic necrosis associated with sorbitol administration.

In a retrospective study, the number needed to cause one colonic perforation was found to be one in 1395 patients treated with Kayexalate. In another systematic review of 58 cases, the colonic necrosis was the most observed gastrointestinal side effect apart from ischemic colitis, perforation, and bleeding. It is, nevertheless, important to know that sodium polystyrene sulfate contains a significant amount of sodium, approx. 100 mg (4.1, Eq) per gram of the drug, which could cause a variable level of sodium retention with incremental dosage or consistent usage leading to severe hypertension or congestive heart failure exacerbation [6].

Abraham et al. studied the clinical, endoscopic, and histologic features of 11 patients with Kayexalate-induced upper gastrointestinal tract damage. The Kayexalate crystals were noted in the biopsy specimen of the esophagus (*n* = 7), stomach (*n* = 6), and duodenum (*n* = 2). In this study, gastrointestinal bleeding (36%) was the common indication for the endoscopic procedure, and endoscopic mucosal abnormality was identified in all 11 patients. Mucosal erosion or ulceration was identified in 82% (9/11) of the patients; one patient experienced Kayexalate with sorbitol-associated esophageal necrosis. 8 out 11 patients had abnormal endoscopic esophageal mucosal appearance, and histologic examination confirmed the presence of the Kayexalate crystals in 7 out 8 patients. The mucosal abnormalities were ranging from mild esophageal erythema, esophageal erosion, esophageal wall thickening, and plaque-resembling candida esophagitis to esophageal mass-like lesion.

Garcia Rodriguez et al. reported a case of acute esophageal necrosis in a patient with the previous history of end-stage renal disease on intermittent hemodialysis therapy, peripheral vascular disease, and liver cirrhosis who was treated with sodium polystyrene sulfonate (SPS; Kayexalate) for hyperkalemia. The patient developed upper gastrointestinal bleeding, subsequently diagnosed to have acute esophageal necrosis on endoscopy. The biopsies from the esophagus showed basophilic “fish scale-like” crystals consistent with SPS resins in a background of an acute inflammatory exudate with necrosis as an etiology of acute esophageal necrosis [7].

It is well documented that Kayexalate causes upper GI (gastrointestinal) tract mucosal damage; however, the degree of mucosal damage or necrosis is more severe in the lower GI tract than the upper GI tract [8]. Emmanuel et al. published a case of nonbleeding esophageal ulcer in a patient who presented with coffee-ground emesis. The histologic findings were consistent with ulcerative esophagitis and the presence of Kayexalate crystals. The patient was taking oral Kayexalate therapy for one week before the onset of the symptoms. Oral Kayexalate was discontinued, and the esophageal ulcer healed with PPI (proton-pump inhibitor) therapy [9]. SPS-induced hemorrhagic duodenitis requiring blood transfusion in association with CMV enteritis was reported by Gurtler et al. in a patient with a history of a kidney transplant [10].

Generally, gastrointestinal complications are more common in critically ill or uremic patients; hence, its use should be reserved for emergent circumstances when other modalities are not immediately available. The diagnosis is usually made by strong clinical suspicion and temporal relation to Kayexalate ingestion followed by endoscopic examination with mucosal biopsies. The pathognomonic histologic findings include basophilic angulated striated crystals in a “fish scale” pattern [11]. Cholestyramine could give rise to similar histologic findings; therefore, it should be excluded by patient history [12]. The treatment of Kayexalate-caused bleeding esophageal ulcer is the discontinuation of Kayexalate therapy and standard management approach of upper gastrointestinal bleeding from any other etiology such as nothing per os (NPO), hemodynamic resuscitation, intravenous proton-pump inhibitor, and endoscopic therapy of actively bleeding lesion or lesion with high-risk bleeding stigmata.

## 4. Conclusion

Upper and lower GI tract side effects from oral or rectal Kayexalate use can range from mild GI upset to lethal sequelae such as GI bleeding, colonic ischemia, colonic necrosis, and perforation. The risk of complication with Kayexalate use is high in patients with underlying comorbid medical conditions and the immediate postoperative period. In our patient, the kayexalate was deemed causative agent for bleeding esophageal ulcer given absence of esophageal ulcer on endoscopy prior to this hospitalization, onset of symptoms immediately after ingestion of kayexalate, and propensity of Kayexalate to cause gastrointestinal ulcer and bleeding.

## Figures and Tables

**Table 1:** Laboratory workup during hospitalization.

Lab results	Normal range	Day 1	Day 4	Day 6
Hemoglobin/hematocrit	12–16 g/dl/42–51%	5.7/16	7.1/21	5.3/15.3
White cell count	4.8–10.8 K/ul	15	9	21
Platelets	150–400 K/ul	54	45	69
Sodium	135–145 mEq/L	140	142	140
Potassium	3.5–5.0 mEq/L	6.1	5.6	4.6
Bicarbonate	24–30 mEq/L	18	16	25
Chloride	98–108 mEq/L	108	105	120
Blood urea nitrogen	6–20 mg/dl	47	30	19
Creatinine	0.5–1.5 mg/dl	3.2	2.6	2.4
Calcium	8.5–10.5 mg/dl	9.2		
Total protein	6.0–8.5 g/dl	5.5	5.7	5.7
Albumin	3.2–4.8 g/dl	2.2	2.7	2.7
Alanine aminotransferase	5–40 U/L	14	15	31
Aspartate aminotransferase	9–36 U/L	43	42	76
Alkaline phosphatase	42–98 U/L	42	39	
Total bilirubin/direct bilirubin	0.2–1.2 mg/dl/0.0–0.3 mg/dl	8.0/3.0	11/4.2	
Prothrombin time	9.5–12 sec	22		
Partial thromboplastin time	26–33 sec	46		
Fibrinogen assay		95		
D-dimer		985		

**Table 2:** Workup of nephrotic syndrome.

Lab results	Normal range	Values
C3 complement serum	90–150 mf/dl	33
C4 complement serum	16–47 mg/dl	13
Rheumatoid factor		Negative
ANCA		Negative
Anti-RNP		Negative
Anti-smooth muscle antibody		Negative
Cryoglobulin		Negative
Urine protein	0–30 mg/dl	1610
Urine creatinine	20–200 mg/dl	246

## Data Availability

No data were used to support this study.
